# Caring for Older Patients With Cancer: Estimates of the Prevalence, Burden, and Unmet Needs of Caregivers in the United States

**DOI:** 10.1093/geroni/igaa057.2412

**Published:** 2020-12-16

**Authors:** Erin Kent

**Affiliations:** University of North Carolina at Chapel Hill, Chapel Hill, North Carolina, United States

## Abstract

In 2020, ~1.8 million Americans are expected to be newly diagnosed with cancer, with approximately 70% of cases diagnosed over the age of 65. Cancer can have a ripple effect, impacting not just patients themselves, but their family caregivers. This presentation will provide an overview of the estimates of the number of family caregivers caring for individuals with cancer in the US, focusing on older patients, from several population-based data sources: Caregiving in the US 2020, the Health Information National Trends Survey (HINTS, 2017-2019), the Behavioral Risk Factors Surveillance System (BRFSS, 2015-2019), and the National Health and Aging Trends (NHATS) Survey. The presentation will compare features of the data sources to give a comprehensive picture of the state of cancer caregiving. In addition, the presentation will highlight what is known about the experiences of cancer caregivers, including caregiving characteristics, burden, unmet needs, and ideas for improving support for family caregivers.

